# Transmission of potato spindle tuber viroid between Phytophthora infestans and host plants

**DOI:** 10.18699/VJGB-22-34

**Published:** 2022-05

**Authors:** O.S. Afanasenko, A.V. Khiutti, N.V. Mironenko, N.M. Lashina

**Affiliations:** All-Russian Research Institute of Plant Protection, Pushkin, St. Petersburg, Russia; All-Russian Research Institute of Plant Protection, Pushkin, St. Petersburg, Russia; All-Russian Research Institute of Plant Protection, Pushkin, St. Petersburg, Russia; All-Russian Research Institute of Plant Protection, Pushkin, St. Petersburg, Russia

**Keywords:** potato; ; ; ; ;, tomato, PSTVd strains, transmission, Phytophthora infestans, RT-PCR detection, картофель; ; ; ; ;, томаты, штаммы ВВКК, трансмиссия, Phytophthora infestans, ОТ-ПЦР-диагностика

## Abstract

Potato spindle tuber viroid (PSTVd) is a naked, circular, single-stranded RNA (356–363 nucleotides in length) which lacks any protein-coding sequences. It is an economically important pathogen and is classified as a high-risk plant quarantine disease. Moreover, it is known that PSTVd is mechanically transmitted by vegetative plant propagation through infected pollen, and by aphids. The aim of this study is to determine the possibility of viroid transmission by potato pathogen Phytophthora infestans (Mont.) de Bary. PSTVd-infected (strain VP87) potato cultivars Gala, Colomba, and Riviera were inoculated with P. infestans isolate PiVZR18, and in 7 days, after the appearance of symptoms, re-isolation of P. infestans on rye agar was conducted. RT-PCR diagnostics of PSTVd in a mixture of mycelia and sporangia were positive after 14 days of cultivation on rye agar. The PSTVd-infected P. infestans isolate PiVZR18v+ was used to inoculate the healthy, viroid-free plants of potato cv. Gala and tomato cv. Zagadka. After 60 days, an amplification fragment of PSTVd was detected in the tissues of one plant of tomato cv. Zagadka by RT-PCR with the primer set P3/P4, indicating successful transmission of PSTVd by P. infestans isolate PiVZR18v+. This result was confirmed by sequencing of the RT-PCR amplicon with primers P3/P4. The partial sequence of this amplicon was identical (99.5 %) to PSTVd strain VP87. RT-PCR showed the possibility of viroid stability in a pure culture of P. infestans isolate PiVZR18v+ after three consecutive passages on rye agar. PSTVd was not detected after the eighth passage on rye agar in P. infestans subculture. These results are initial evidence of potato viroid PSTVd being bidirectionally transferred between P. infestans and host plants

## Abstract (ru)

Вироид веретеновидных клубней картофеля (ВВКК) представляет собой кольцевую одноцепочечную РНК длиной 356–363 нуклеотида, в которой отсутствуют какие-либо последовательности, кодирующие белок. ВВКК является экономически значимым заболеванием картофеля, имеющим статус карантинного. Известно, что ВВКК передается механически при вегетативном размножении растений, через инфицированную пыльцу и с помощью тлей. Целью данного исследования было определение возможности передачи ВВКК (штамм VP87) от растений картофеля и томата, зараженных вироидом, патогену Phytophthora infestans (Mont.) de Bary и от P. infestans – растениям картофеля и томата. Cорта картофеля Гала, Коломба и Ривьера, инфицированные ВВКК, инокулировали изолятом P. infestans PiVZR18; через 7 дней после появления симптомов фитофтороза провели повторное выделение P. infestans в чистую культуру на ржаной агар. Через 14 дней культивирования P. infestans на ржаном агаре в смеси мицелия и спорангиев методом ОТ-ПЦР был обнаружен ВВКК. ВВКК-инфицированным изолятом P. infestans (PiPSTVdv+) провели инокуляцию растений томата сорта Загадка и растений картофеля сорта Гала. Через 60 дней в листьях томата сорта Загадка методом ОТ-ПЦР с праймерами Р3/Р4 был выявлен диагностический продукт амплификации 360 п. о., свидетельствующий об успешной трансмиссии ВВКК изолятом PiVZR18v+. Результаты были подтверждены секвенированием продукта амплификации ВВКК. Нуклеотидная последовательность вироида в растении томата, зараженном изолятом PiVZR18v+, оказалась на 99.5 % идентичной использованному в эксперименте штамму VP87. Для доказательства возможности сохранения вироида в чистой культуре P. infestans изолят PiVZR18v+ пассировали на ржаном агаре с интервалом 30 дней. После трех последовательных пассажей на ржаном агаре ВВКК был диагностирован в культуре изолята, что подтверждено секвенированием продукта амплификации с вироид-специфичными праймерами. В субкультуре P. infestans после восьмого пассажа на ржаном агаре ВВКК не обнаруживался. Полученные данные свидетельствуют о двунаправленной передаче ВВКК в патосистеме P. infestans – растение-хозяин.

## Introduction

Potato spindle tuber viroid (PSTVd) is an economically important pathogen, classified as a high-risk plant quarantine disease. According to the European Plant Protection Organization (EPPO), the disease has been reported in 37 countries on all continents (https://gd.eppo.int/taxon/PSTVD0/ distribution). In Russia, and other former Soviet Union regions, PSTVd was detected in about 50–70 % of in vitro potato plants (Kastalyeva et al., 1992).

Natural infections of PSTVd have been found in the field, mainly in solanaceous crops, such as pepino (Puchta et al., 1990), potato (Diener, Raymer, 1969), and tomato (Puchta et al., 1990). Potato plants infected with PSTVd become smaller and show leaf yellowing, and infected tubers become smaller and cracked. The reduction in tuber weight depends on the viroid strain, potato cultivar, and length of time they have been infected with PSTVd (Pfannenstiel, Slack, 1980). Furthermore, a reduction in tuber yield of up to 24 % has been reported in cultivar Saco infected with mild strains of PSTVd, however, severe strain reduced the yield by up to 64 % (Singh R.P., 1970). In addition to direct losses, it is important to take into account indirect losses that can be significant due to the quarantine status.

PSTVd belongs to Family Pospiviroidae (1POSPF), Genus Pospiviroid (1POSPG) and consists of a naked, circular, single-stranded RNA (356–363 nucleotides in length) – the smallest among plant pathogens lacking a protein-coding ability – therefore, it is a parasite of the host transcription mechanism (Yanagisawa et al., 2019).

PSTVd has a wide host range of at least 138 species across 10 families (Singh R.P., 1973). The main hosts are from the Solanaceae family (Owens et al., 1992; Mertelik et al., 2010; Mackie et al., 2016). PSTVd is transmitted mechanically (Verhoeven, Roenhorst, 2010), by aphids (Syller et al., 1997). Moreover, it was found to be vertically transmitted through pollen to progeny seeds on potato (Solanum tuberosum) and tomato (S. lycopersicum) and horizontally transmitted through infected pollen to other potato and tomato plants (Kryczyński et al., 1988; Singh R.P. et al., 1992; Matsushita, Yanagisawa, 2018).

Viruses are common in fungi and oomycetes and some of these viruses share sequence identities with plant viruses belonging to different families and genera (Mascia et al., 2019). There are several examples of plant–virus transmission by phytopathogenic fungi and oomycetes. It was shown that soil-inhabiting fungi Olpidium brassicae and O. radicale belonging to Chytridiales and Polymyxa graminis, Spongospora subterranean, and Synchytrium endobioticum – belonging to the order Plasmodiophorales – transmit plant viruses (Bhat, Rao, 2020). Replications of the tobacco mosaic virus were demonstrated in the phytopathogenic fungi Colletotrichum acutatum, C. clavatum, and C. theobromicola (Mascia et al., 2019), cucumber mosaic virus was reported in Rhizoctonia solani (Andika et al., 2017), artichoke Italian latent virus, artichoke mottled crinkle virus, potato virus X, potato virus Y, tobacco mosaic virus and cucumber mosaic virus plus its satellite RNA can replicate and persist in Phytophthora infestans at least through the first subculture (Mascia et al., 2019).

Wei et al. (2019) obtained preliminary data on the possibilities of replicating hop stunt viroid (HSVd), iresine 1 viroid belonging to the Pospiviroidae and avocado sunblotch viroid (Avsunviroidae) in at least one of phytopathogenic ascomycete fungi Cryphonectria parasitica, Valsa mali, and Fusarium graminearum

Оomycete P. infestans causes significant losses to potato and tomato crops on a global scale. Despite intensive use of fungicides, the pathogen is constantly and ubiquitously present in potato crops. P. infestans is also the most harmful and widespread tomato pathogen, both in field and greenhouse conditions. This oomycete has a high adaptive potential to the host plants, which may indicate the formation of competitive relationships with other potato and tomato pathogens. In this regard, it is of interest to identify a possible role of P. infestans in transmission of PSTVd to potato and tomato plants.

## Materials and methods

Plant materials. Potato cultivars that, according to our data, were susceptible to both PSTVd strain VP87 and P. infestans – Gala, Riviera, and Colomba and tomato cultivars Zagadka, Moskvich, and Damskiy Palchik were included in the study.

These potato and tomato cultivars were registered in the Russian State Register of Breeding Achievements.

PSTVd strains. Two intermediate PSTVd strains, VP35 (GenBank accession no. LC523658) and VP87 (LC523667), and severe strain FP10-13 (LC523676) deposited in the international information database DDBJ (DNA Data Bank of Japan), Data set “Viruses” (http://blast.ddbj.nig.ac.jp/) were used in the study. These strains were isolated from infected potato leaves from the Volga Federal District (VP87 and VP35) and tubers from the Far Eastern Federal District (FP10-13) in 2019 (Matsushita et al., 2021).

The PSTVd strains were supported on living tomato plants of Russian cultivars Zagadka, Moskvich, and Damskiy Palchik.

Isolate of P. infestans. Isolate PiVZR18 of P. infestans was used in the experiments on viroid transmission. PiVZR18 was isolated from the natural population of P. infestans in the Leningrad Region (northwest of the European part of Russia) in 2018. Eight virulence genes (1, 2, 3, 4, 6, 7, 10, and 11) were identified in this isolate on a set of Black’s differentials (Black et al., 1953).

Viroid inoculation of plants. Potato and tomato plants were grown in a growth room at a temperature of 25 °C with a photoperiod of 16h/8h (day/night) in 2l pots filled with “Terra vita” soil. Seven-day germination potato plants and 14-day tomato plants were used for inoculation by PSTVd.

To prepare the inoculum, 0.1 g of fresh tomato leaf tissue – 60 days post inoculation (dpi) with PSTVd strain VP87 – was ground in 1 ml sodium phosphate buffer (pH 7.0) and filtered through cheesecloth

For mechanical inoculation, the cotyledons of tomato were dusted with carborundum and gently rubbed over the surface of the leaves with a plastic pestle. Ten microliters of inoculum was placed on the injured leaf surface and rubbed several times with a sterile plastic pestle. The inoculated plants were incubated for two months at 25 °C with light intensity (fluorescent, 40 W, ×4).

At 60 dpi, the presence of viroid in the inoculated tomato plants was determined by RT-PCR.

To inoculate 7-day potato plants of the cultivars Gala, Riviera, and Colomba, a 0.5–1.0 cm longitudinal stem incision was performed with a sterile razor on a stem apex (Suppl. Fig. 1)1, and 10 μl of the PSTVd VP87 strain suspension – obtained as described above – was applied. Three plants of each potato cultivar were inoculated and the assay was repeated three times. In 60 dpi, the presence of PSTVd in the inoculated plants of potato cultivars was determined by RT-PCR.

Supplementary Materials are available in the online version of the paper: http://www.bionet.nsc.ru/vogis/download/pict-2022-26/appx5.pdf/


P. infestans inoculation of plants. Isolate PiVZR18 of P. infestans was cultured on rye agar medium (1.0 Li ddH2O, 60.0 g rye organic berries (grind in blender), 20.0 g sucrose, 15.0 g agar) for 30 days in the dark at 15 °C for propagation and morphological observation (Medina, Platt, 1999).

Before inoculating the plants, the suspension was incubated at 12 °C for 2.5–3 h to release zoospores. Upon RT-PCR detection for viroid infection, both healthy and viroid-infected tomato and potato plants were inoculated with a suspension of P. infestans at a concentration of 50.000 zoosporangia in 1 ml. After inoculation, the plants were placed in humid chambers with a 14 h light period, at 23 °C during the day and 15 °C at night for a period of 13 dpi. To study PSTVd transmission from P. infestans to host plants, when typical symptoms of late blight appeared, the humid chamber was removed and the development of P. infestans slowed down. The affected leaves were removed and the plants continued to grow. For the purposes of PSTVd diagnostics, the upper leaves of the plants without late blight symptoms were cut off.

Viroid inoculation of P. infestans. Inoculum of PSTVd was obtained from the infected tomato plants as described above and applied to the 14-day P. infestans culture by transferring 10 μl per Petri dish (in the dish center). After inoculation, the culture was left to grow for 15 days at 10 °C. Then, mycelia from the periphery and from the center of the colony were transferred separately to a fresh medium. The culture was left in the same conditions for 30 days, after which RT-PCR analysis was conducted.

Isolation of P. infestans from infected potato and tomato plants. Seven days post inoculation (dpi) after the symptoms of late blight appeared, P. infestans was isolated from the plants. Sections of the infected leaves were placed between tuber slices of the healthy cv. Colomba, and at 6 days, mycelium was transferred with a needle to the surface of rye agar. The isolates were cultured for 30 days at 15 °C in the dark and then transferred to a fresh medium.

Viroid detection and sequencing. We collected the uppermost leaves from the inoculated potato or tomato plants at 60 dpi. PSTVd detection in pure culture of P. infestans was carried out after 30 days of growing on rye agar. Approximately 0.1 g of tissue from leaves or mycelium was used for RNA extraction. Total RNA was extracted using the RNeasy Plant Mini Kit (Qiagen, Hilden, Germany) as per the manufacturer’s instructions (http://www.genome.duke.edu/cores/microarray/ services/rna-qc/documents/RNeasy_Mini_Handbook.pdf) and subsequently used for one-step RT-PCR. Primer sets P3/P4 (Behjatnia et al., 1996) and P1/P2 (Gross et al., 1978) or 68PV-R+87PV-F (Yanagisawa et al., 2019) were used to detect PSTVd. RT-PCR was prepared with the PrimerScript One-Step RT-PCR Kit ver 2 reagents in 10 ml (Takara Bio Inc., Shiga, Japan) following the manufacturer’s instructions. The primer set ITS4/ITS5 (White et al., 1990; Ristaino et al., 1998) was used to detect the ITS region of P. infestans as an internal control.

RT-PCR was carried out on a MyCycler Thermal Cycler (Bio-Rad, California, USA) at 50 °C for 30 min, 94 °C for 2 min, followed by 35 cycles of 94 °C for 30 sec, 60 °C for 30 sec and 72 °C for 30 sec. An additional elongation step was performed at 72 °C for 5 min followed by storage at 12 °C. The sizes of the diagnostic fragments of PSTVd and the ITS region were 360 and 946 bp, respectively.

The PSTVd amplicons were sequenced at the Beagle Company (St. Petersburg, Russia). Alignment and manual editing of nucleotide sequences were performed using Vector NTI Advance 10 software (Thermo Fisher Scientific). The obtained nucleotide sequences were tested for similarity with PSTVd strain VP87 (LC523667), used in this study, deposited in the international information database DDBJ (DNA Data Bank of Japan), data set “Viruses” (http://blast. ddbj.nig.ac.jp/).

## Results

There is an absence of data on the possibility of replicating PSTVd in P. infestans and bidirectionally transferring it between host plants and P. infestans. In this study, we investigated the possibility of PSTVd transmission (1) from host plants to P. infestans, (2) from P. infestans to host plants, and (3) the possibility of PSTVd stability in pure cultures of P. infestans.

Transmission of PSTVd from host plants to P. infestans

**Fig. 1.**
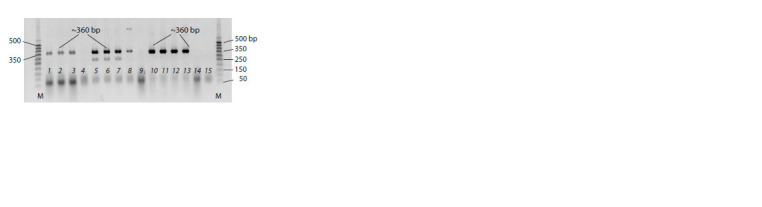
RT-PCR detection of PSTVd with the primer set P3/P4 in leaves of infected potato plants by PSTVd strain VP87 before inoculation with P. infestans. 1–3, Individual plants of cv. Riviera; 4, mock-inoculated cv. Riviera; 5–8, individual plants of cv. Colomba; 9, mock-inoculated cv. Colomba; 10–13, individual plants of cv. Gala; 14, mock-inoculated cv. Gala; 15, negative control (distilled water); M, molecular weight marker 50 bp DNA ladder (Primetech DNA Ladder).

From potato plants. Upon confirming PSTVd infection in tomato plants of cv. Zagadka by RT-PCR, inoculation of three potato cultivars (Gala, Colomba, and Riviera), using as an inoculum source extracted-sap of tomato infected with PSTVd strain VP87, was conducted (see Suppl. Fig. 1). After 60 days, detection of PSTVd presence in plants of these cultivars was carried out by RT-PCR with the P3/P4 primer set (Fig. 1). The brightest amplicons indicating a high accumulation of the viroid were found for cv. Gala and cv. Colomba. The viroid accumulation was lower in three plants of cv. Riviera (see Fig. 1).

**Fig. 2.**
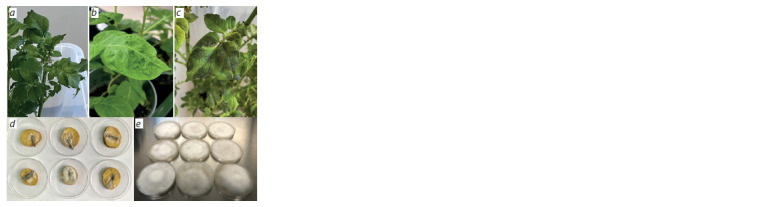
PSTVd transmission from potato to P. infestans a, Mock-inoculated (distilled water) potato cv. Colomba; b, PSTVd-infected (strain VP87) potato cv. Colomba; c, symptoms of P. infestans on PSTVd-infected potato cv. Colomba (strain VP87); d, tuber slices of healthy cv. Colomba inoculated with P. infestans from the infected plants; e, P. infestans isolates from the PSTVd-infected plants of cv. Colomba on rye agar.

PSTVd-infected and uninfected (control) potato plants were inoculated with P. infestans isolate PiVZR18. Seven days after the appearance of symptoms (Fig. 2, a) caused by P. infestans, the pathogen was first re-isolated from these cultivars on tuber slices of healthy cv. Colomba and then transferred to pure culture on rye agar (see Fig. 2).

**Fig. 3.**
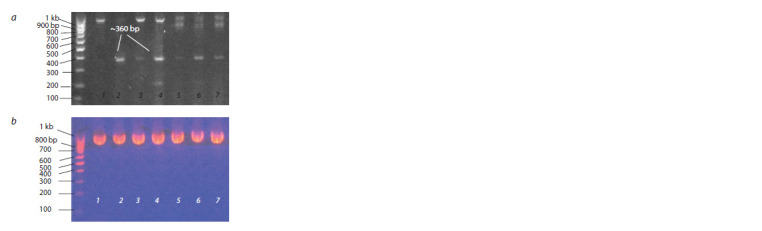
RT-PCR detection of PSTVd in cultures of P. infestans isolated from potato plants infected with the PSTVd strain VP87 (a) and control of P. infestans RNA presence (b). a, RT-PCR detection with the primer set P3/P4; b, RT-PCR amplification of the same RNA samples with the primer set ITS4/ITS5. Lines: 1, PSTVd-uninfected P. infestans isolate PiVZR18; 2, P. infestans isolates from PSTVd-infected potato cv. Gala; 3, 4, from cv. Riviera; 5–7, from cv. Colomba. Left line: molecular weight marker 100 bp (Gene Ruller, Fermentas).

After culturing of P. infestans isolates on rye agar, PSTVd detection by RT-PCR was conducted with the primer set P3/P4 (Fig. 3, a). Amplicons of ~360 bp – indicating the presence of PSTVd – were detected in a mixture of mycelium and sporangia of pure culture of P. infestans isolates after colonization on the cultivars Gala, Riviera, and Colomba (see Fig. 3, a). To control for the negative results of viroid detection that are not due to the quality of RNA extracted from P. infestans samples, we used PCR with universal primers ITS4/ITS5 on rDNAs, which are species specific for P. infestans and displayed an amplicon of 946 bp (Ristaino et al., 1998) (see Fig. 3, b).

**Fig. 4.**
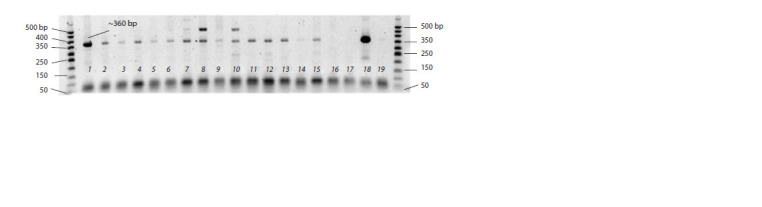
RT-PCR detection of PSTVd with the primer set P3/P4 in P. infestans isolates grown on slices of potato cv. Colomba tubers after their isolation from infected with viroid tomato plants. 1, 2, Isolates from tomato cv. Moskvich infected by PSTVd strain VP87; 3, 4, isolates from tomato cv. Damskiy Palchik, infected by PSTVd strain VP87; 5–9, isolates from tomato cv. Moskvich, infected by PSTVd strain FP10-13; 10–13, isolates from tomato cv. Moskvich, infected by PSTVd strain VP35; 14, 15, isolates from tomato cv. Damskiy Palchik, infected by PSTVd strain VP35; 16, isolate PiVZR18 of P. infestans uninfected by PSTVd (negative control); 17, cv. Colomba potato tuber (negative control); 18, tomato cv. Moskvich infected by PSTVd strain VP87 (positive control); 19, distilled water (negative control). On the right and left sides of the gel, 50 bp DNA ladders (Primetech DNA Ladder) are shown.

From tomato plants. The 15 plants of the two tomato cultivars (Moskvich and Damskiy Palchik) infected with three PSTVd strains (VP87, FP10-13, and VP35) were inoculated with the P. infestans isolate PiVZR18 to confirm the results obtained. Seven days after the appearance of the symptoms caused by P. infestans, 15 cultures of the pathogen were re-isolated from these plants, first on tuber slices of healthy cv. Colomba. After mycelial overgrowth on the surface of tuber slices, the first detection of viroid presence in the mycelium was performed. Out of 15 isolates, the brightest fragment indicating viroid infection of mycelium was detected with the primer set P3/P4 in the P. infestans isolate from cv. Moskvich infected with PSTVd strain VP87 (Fig. 4, line 1). Positive PSTVd detection was also obtained for cv. Damskiy Palchik, infected by PSTVd strain VP87 (line 4) and for the isolates from cv. Moskvich infected by PSTVd strain FP10-13 (lines 6–8) and by strain VP35 (lines 10–13). Weak amplicons were obtained in lines 3, 5, 9, 14, which indicates a low initial concentration of PSTVd in P. infestans mycelium obtained from the host plant (see Fig. 4).

PSTVd transmission from P. infestans to host plants

The PSTVd-infected P. infestans isolate PiVZR18v+ was used to inoculate the healthy, viroid-free plants of potato cv. Gala and tomato cv. Zagadka.

**Fig. 5.**
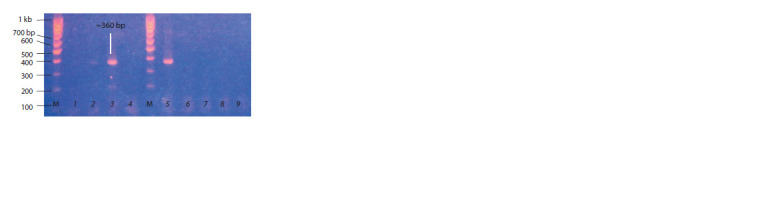
RT-PCR detection of PSTVd with the primer set P3/P4 in potato and tomato plants 60 dpi with viroid-infected P. infestans isolate PiVZR18v+. 1, Potato cv. Gala; 2–4, tomato cv. Zagadka; 5, tomato cv. Zagadka infected by PSTVd strain VP87 (positive control); 6, potato cv. Gala uninfected by PSTVd (negative control); 7, tomato cv. Zagadka uninfected by PSTVd (negative control); 8, PSTVd-uninfected P. infestans isolate PiVZR18 (negative control); 9, distilled water (negative control); M, molecular weight marker 100 bp (Gene Ruller, Fermentas).

After 60 days, an amplification fragment of PSTVd was detected in the tissues of one plant of tomato cv. Zagadka by RT-PCR with the primer set P3/P4, indicating successful transmission of PSTVd by P. infestans isolate PiVZR18v+ (Fig. 5).

The detection of PSTVd in the RNA of cv. Zagadka inoculated with the P. infestans isolate PiVZR18v+ was confirmed by sequencing of the RT-PCR amplicon with primers P3/P4 (see Fig. 5, line 3). The partial sequence (204 bp) of this amplicon was identical (99.5 %) to PSTVd strain VP87 (LC523667) (Suppl. Fig. 2).

Stability of PSTVd in pure culture of P. infestans

**Fig. 6.**
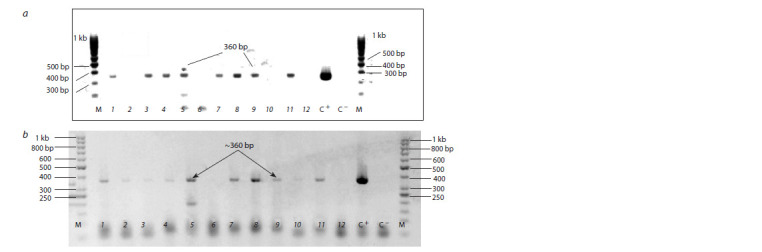
RT-PCR detection of PSTVd with primer sets P3/P4 (a) and 68PV/87PV (b) in cultures of P. infestans isolates after colonization on PSTVd infected potato cv. Colomba. 1–4, Second passage on rye agar; 5–11, third passage on rye agar; 12, PSTVd-uninfected isolate PiVZR18 (negative control); C+, cv. Colomba infected by PSTVd (positive control); C–, distilled water; М, molecular weight marker 100 bp (a) and 50 bp (b) (Gene Ruller, Fermentas).

**Fig. 7.**
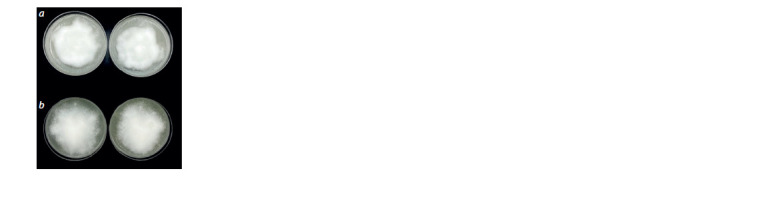
Cultures of P. infestans PiVZR18: a, infected with viroid after three passages on rye agar: b, healthy.

The stability of strain VP87 in pure culture of P. infestans isolate PiVZR18v+ after consecutive passages on rye agar was studied. RT-PCR with primer sets P3/P4 (Fig. 6, a) and 68PV/87PV (Fig. 6, b) revealed amplification products indicating the presence of PSTVd after the second and third passages on rye agar of P. infestans isolates from viroidinfected cv. Colomba (see the Table, Fig. 6). PSTVd stability in P. infestans isolates after three passages on rye agar was shown by sequencing of the RT-PCR amplicon with primers P3/P4. The partial sequence of RT-PCR amplicon (near 232 bp) of viroid in P. infestans isolate PiVZRv+ after the third passage on rye agar received with the primer set P3/P4 is identical (98.3 %) to PSTVd strain VP87 (LC523667). Another partial sequence of RT-PCR amplicon (270 bp) received with the primer set 68PV/87PV of the same RNA sample is identical to PSTVd strain VP87 (LC523667) – 99.3 % (Suppl. Fig. 3). On the other hand, PSTVd was not detected after the eighth passage on rye agar in P. infestans subculture (see the Table). P. infestans isolates infected with viroid strain VP87 were characterized by more abundant, but also more compact mycelium, forming an almost felt-like colony (Fig. 7).

**Tab:**
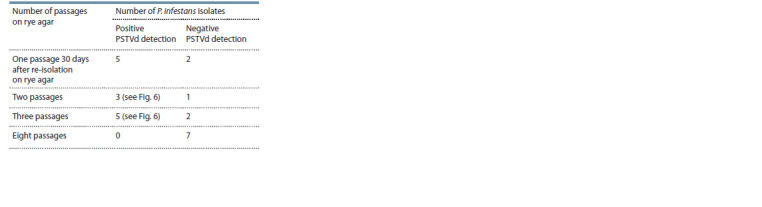
Presence of amplification product 360 bp in P. infestans isolates after colonization on viroid-infected plants of potato cv. Colomba (each passage is 30 days)

**Fig. 8.**
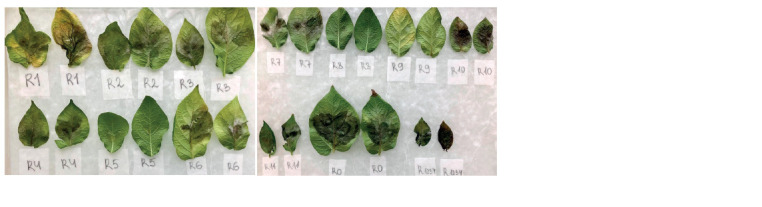
Virulence of the PSTVd-infected PiVZR18v+ to the lines of a set of Black’s differentials (11 resistance genes). Eight virulence genes (1, 2, 3, 4, 6, 7, 10, 11) were determined.

**Fig. 9.**
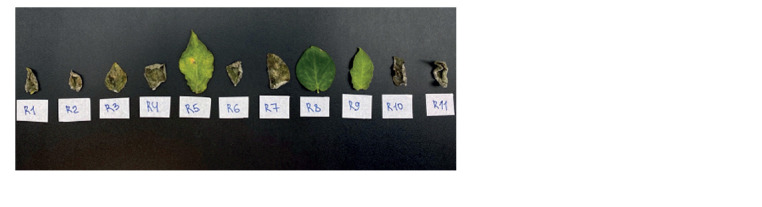
Virulence of the initial PSTVd-uninfected P. infestans PiVZR18 isolate to the lines of a set of Black’s differentials (11 resistance genes). Eight virulence genes (1, 2, 3, 4, 6, 7, 10, 11) were determined.

Virulence testing on a set of Black’s potato differentials (11 lines with different resistance genes) (Black et al., 1953) of the PSTVd-infected PiVZR18v+ and the initial uninfected PiVZR18 isolates showed the same types of reactions on detached leaves of 11 lines after 7 dpi (Fig. 8, 9).

For both isolates, eight virulence genes (1, 2, 3, 4, 6, 7, 10, and 11) were identified. The PSTVd-infected P. infestans isolate seems to be less aggressive in comparison to the uninfected isolate; however, this observation requires further study.

## Discussion

Potato (Solanum tuberosum) is one of the most important staple crops worldwide. According to the FAO, over 390 million tons are produced on over 19 million ha of farmland worldwide (http://www.fao.org/faostat/en/#data/QCL). The quarantine status of potato tuber spindle viroids and possible significant losses of potato yield determine the importance of studying various aspects of pathogen epidemiology.

PSTVd replication is accompanied by the accumulation of viroid-derived small RNAs (vd-siRNAs) suggested to play a central role in disease symptom development

Potatoes were domesticated in the Andes in Southern Peru around 10,000 years ago. Nevertheless, the introduction of the potato to Europe, along with all its associated diseases, dates back to the 16–17th centuries (Khavkin, 2015). Late blight of potato and tomato is a devastating disease caused by funguslike oomycete P. infestans. Despite many efforts, the severity of this disease has increased dramatically in recent years.

It is known that the mode and speed of spreading plant pathogens are the major factors in the development of epiphytotics. Early studies showed that PSTVd is spread primarily through the use of infected plant material produced vegetatively or as botanical seeds (Fernow et al., 1970), through mechanical spreading across the growing crop, particularly between plants of different species of the Solanaceae family (Manzer, Merriam, 1961; Verhoeven, Roenhorst, 2010). The success of mechanical transmission depends on infected host plant species or cultivars, as well as the frequency and severity of the disease and the temperature (Bulletin OEPP/EPPO Bulletin, 2011). Importantly, transmission to potato and other test plants by aphids (Myzus persicae) was successful only if PSTVd RNA was encapsidated by potato leafroll virus (PLRV) particles (Salazar et al., 1995; Querci et al., 1997).

Some viruses are spread by vectors, which can include pathogenic fungi (Andika et al., 2017; Sutela et al., 2019), oomycetes (Mascia et al., 2019), and nematodes (Brown et al., 1989; Singh S. et al., 2020). Bidirectional transfer between Fusarium graminearum and tobacco plants of hop stunt viroid (HSVd) during infection was shown by Wei et al. (2019). However, Nicotiana benthamiana is not a natural host for either HSVd or F. graminearum. Given this fact (Serra et al., 2020), more evidence is needed to validate the possibility of viroid transmission by phytopathogenic fungi. We showed the presence of PSTVd in P. infestans isolates after colonization on plants of three potato cultivars infected with viroid. After three passages on rye agar (30 days each), RT-PCR analysis showed the presence of viroid in pure cultures of P. infestans. The partial RT-PCR amplicon sequence of viroid in P. infestans isolate PiVZRv+ after the third passage on rye agar is identical (98.3–99.3 %) to PSTVd strain VP87 that was used for the initial inoculation.

Sixty days after inoculation of healthy tomato plants with P. infestans isolate carrying PSTVd, RT-PCR revealed a 360 bp amplification product, indicating successful infection of the plants. This is the first report of horizontal transfer of potato viroid PSTVd between P. infestans and host plants.

Moreover, there is evidence that small RNAs (sRNAs, approximately 20–30 nt) can horizontally transfer from microbes to plants and spread silencing information toward the targeted genes (Han, Luan, 2015). Small RNAs were also found in fungi (Wang, Dean, 2020) and fungal-like Oomycota (Jahan et al., 2015). In addition, sRNAs of 19–40 nt were found from P. infestans (Vetukuri et al., 2012). There are numerous reports of sRNA cross-transfer between plants and pathogens (Zeng et al., 2019; Wang, Dean, 2020). sRNAs can be transported within an organism through the inner side of the plasma membrane (symplast), or cell wall (apoplast) (Wang, Dean, 2020). It is suggested that sRNAs are translocated by extracellular vesicles (EVs) from Arabidopsis to P. capsica (Hou et al., 2019) and B. cinerea (Cai et al., 2018). Furthermore, it is possible that interaction between oomycete and potato involves not only sRNA exchange but also the movement of larger viroid RNA molecules from mycelium into a plant and vice versa.

PSTVd replicates in the nucleus, traffics long distances in the phloem, and moves cell-to-cell via plasmodesmata in plants (Takeda, Ding, 2009). After the third subculture, PSTVd was detected from P. infestans, suggesting that PSTVd can replicate in the nucleus and locate to non-septate hyphaе of P. infestans (see the Table). On the other hand, after the eighth subculture, PSTVd accumulation in P. infestans was not detected by RT-PCR. The same results were obtained by Wei et al. (2019), in which PSTVd was eliminated from Cryphonectria parasitica, Valsa mali, and Fusarium graminearum after eight subcultures. This disappearance could be caused by a defense mechanism against viroid, namely, the RNA silencing system. Viroids are the target of the RNA silencing system and become elicitors of the host defense system via RNA silencing (Cottilli et al., 2019; Wei et al., 2020). Thus, PSTVd could have been degraded by the silencing system, resulting in the elimination of PSTVd from P. infestans

Phytophthora infestans produce sporangia on the surface of potato leaves, and then zoospores, released from sporangia, form walled cysts on the plant surface (Mazumdar et al., 2021). The cysts germinate and extend a germ tube into the leaves and stems of the host plants. PSTVd transferred from P. infestans to plants, suggesting that PSTVd was present not only in mycelium but also in sporangia and zoospores. Mature sporangia were dispersed by wind or water (Leesutthiphonchai et al., 2018). Thus, there is a possibility that PSTVd can spread long distances via infected sporangia. In contrast, there is still no evidence of viroid infection in isolates of P. infestans from field populations and the possibility of viroid stability in the mycelium of P. infestans in tubers is unclear.

Concerning mycoviruses, there are two hypotheses of their origin: the first states that they are of an unknown but ancient origin and have coevolved along with their hosts, the second one suggests they have relatively recently moved from a fungal plant host into fungus (Pearson et al., 2009). Both hypotheses are also applicable to PSTVd. Prolonged coexistence of viroid–P. infestans–host plants can lead to viroid transition from a host plant to an oomycete.

## Conclusion

Potato spindle tuber viroid is known as autonomously replicating pathogen only of plants and mainly of solanaceous crops, that lacks any protein-coding sequences. Herein, we demonstrate the possibility of viroid transmission from host plants (potato and tomato) to Phytophthora infestans, from P. infestans to host plants, and the possibility of PSTVd stability in pure cultures of P. infestans after three consecutive passages on rye agar. These results are initial evidence of bidirectionally transferred potato viroid PSTVd between P. infestans and host plants.

## Conflict of interest

The authors declare no conflict of interest.
